# Venous thoracic outlet syndrome and hemodialysis

**DOI:** 10.3389/fsurg.2023.1149644

**Published:** 2023-03-22

**Authors:** Mark G. Davies, Joseph P. Hart

**Affiliations:** ^1^ Center for Quality, Effectiveness and Outcomes in Cardiovascular Diseases, Division of Vascular and Endovascular Surgery, University of Texas Health at San Antonio, San Antonio, TX, United States; ^2^ Division of Vascular and Endovascular Surgery, Medical College of Wisconsin, Milwaukee, WI, United States

**Keywords:** thoracic outlet syndrome, hemodialysis, venous, therapy, outcomes

## Abstract

Central venous stenotic disease is reported in 7%–40% of patients needing a central venous catheter for dialysis and in 19%–41% of hemodialysis patients who have had a prior central venous catheter. Half of these patients will be asymptomatic. Venous Thoracic Outlet syndrome in hemodialysis (hdTOS) is part of this spectrum of disease. The extrinsic mechanical compression of the subclavian vein at the costoclavicular triangle between the clavicle and 1st rib results in an area of external compression with a predisposition to intrinsic mural disease in the vein. The enhanced flow induced by the presence of a distal arteriovenous access in all patients exacerbates the subclavian vein’s response to ongoing extrinsic and intrinsic injury. Repeated endovascular interventions during the maintenance of vascular access accelerates chronic untreatable occlusion of the subclavian vein in the long term. Similar to patients with central venous stenosis, patients with hdTOS can present immediately after access formation with ipsilateral edema or longitudinally with episodes of access dysfunction. hdTOS can be treated in an escalating manner with arteriovenous access flow reduction to <1,500 ml/min, endovascular management, surgical decompression by first rib resection in healthy patients and medial clavicle resection in less healthy patients followed by secondary venous interventions, or finally, a venous bypass. hdTOS represents a complex and evolving therapeutic conundrum for the dialysis community, and additional clinical investigations to establish robust algorithms are required.

## Introduction

Central venous obstructive disease represents a continuing challenge to the maintenance of dialysis access in patients with end stage renal disease. Central venous stenotic disease is reported in 7%–40% of patients needing a central venous catheter for dialysis and in 19%–41% of hemodialysis patients who have had a prior central venous catheter (1). Half of these patients will be asymptomatic before placement of an ipsilateral arteriovenous access site. However, central venous stenosis encompasses stenosis of all veins within the thoracic cavity and deciphering the proportion directly related to Venous Thoracic Outlet Syndrome in hemodialysis (hdTOS) has yet to be effectively reported. The aim of this review is to examine the current state of the art for thoracic outlet syndrome in patients undergoing hemodialysis.

## Anatomy

In hemodialysis, a patient’s own veins or an alternative bridging conduit are used to create an arterio-venous conduit that provides a high-flow, low-pressure circuit for dialysis access. All veins in the arm lead to the subclavian vein, and the presence of AV access induces high flow in the subclavian vein. High flow leads to dilatation and wall thickening. The subclavian vein traverses a short and narrow anatomical plane between the clavicle and 1st rib resulting in a potential area for physiological and pathological compression termed the costoclavicular triangle (2). This narrow anatomical space leads to physiological compression with arm movements, functional compression without symptoms, and symptomatic compression leading to arm symptoms and often access malfunction.

## Pathophysiology

The extrinsic mechanical compression of the subclavian vein at the junction of the costoclavicular triangle between the clavicle and 1st rib (CCJ) predisposes the patient to intrinsic mural disease within the vein. Unlike classical venous TOS, the enhanced flow induced by the presence of a distal arteriovenous access in all patients and the presence of or a history of a central venous catheter in many patients exacerbates the subclavian vein’s response to ongoing extrinsic and intrinsic injury (3). Patients with pacemaker and defibrillator leads present an additional element of obstruction that compromises resting cross-sectional area. Static and dynamic stenoses of the subclavian vein result and can lead to intraluminal thrombosis. Repeated endovascular interventions accelerate chronic, untreatable obstructive disease of the subclavian vein in the long term because the underlying anatomic compromise is not addressed (4).

## Presentation

Patients with a patent access site and central venous stenosis can present in several ways and many of these symptoms can mimic patients with stenosis solely due to CCJ compression (hdTOS):

*Asymptomatic:* On routine duplex imaging or venography CCJ compression is seen or is induced by TOS position of the arm. The patient does not have symptoms and dialysis access is unaffected.
*Arm Swelling:* Patients may experience upper arm and forearm swelling due to venous hypertension as a result of the higher pressures and high flow induced by the AV access. which may be so significant as to effect arm function. Sudden onset of arm swelling may indicate subclavian vein deep venous thrombosis. Imaging will confirm CCJ compression or stenosis.
*Arm Pain:* Patients may experience arm pain with or with arm swelling, which may be exacerbated while on hemodialysis. Imaging will confirm CCJ compression or stenosis.
*Arterio-venous Access Malfunction:* The access may have increased pressures in the venous circuit or present with thrombosis leading to an inability to obtain effective dialysis. Imaging will confirm CCJ compression or stenosis.

## Imaging

Due to the fact that hdTOS can overlap central venous stenosis in presenting symptoms, an array of imaging modalities of the thoracic outlet in hemodialysis have been described to examine the subclavian vein and the anatomy of the thoracic outlet (5–7). It is important to interrogate the thoracic outlet to confirm or exclude/the presence of a pathological narrowing at the thoracic outlet in the presence of a failure of primary intervention of the central venous stenosis. The simplest is a plain x-ray to determine if there is a cervical rib or another skeletal abnormality. Plain radiographs may also demonstrate fractures in prior placed venous stents. Duplex imaging with provocation can demonstrate venous stenosis that is static or that is induced by provocation maneuvers. Duplex imaging can also evaluate the status of a prior intervention (angioplasty or stenting). Cross-sectional imaging with contrast can be used to assess the thoracic outlet and provide intramural and extramural details on the luminal compromise and/or compression present. Contrast Venography with or without intravascular ultrasound (IVUS) can be performed independently or in combination with a contrast fistulogram to assess the outflow tract of the AV access. Venography allows for dynamic imaging of the subclavian vein with the ability to maneuver the ipsilateral arm to induce physiological or pathological compression. The use of intravascular ultrasound allows for a determination of the presence of intraluminal webs in addition to stenosis with or without provocation.

## Indications for intervention

**Figure 1:**
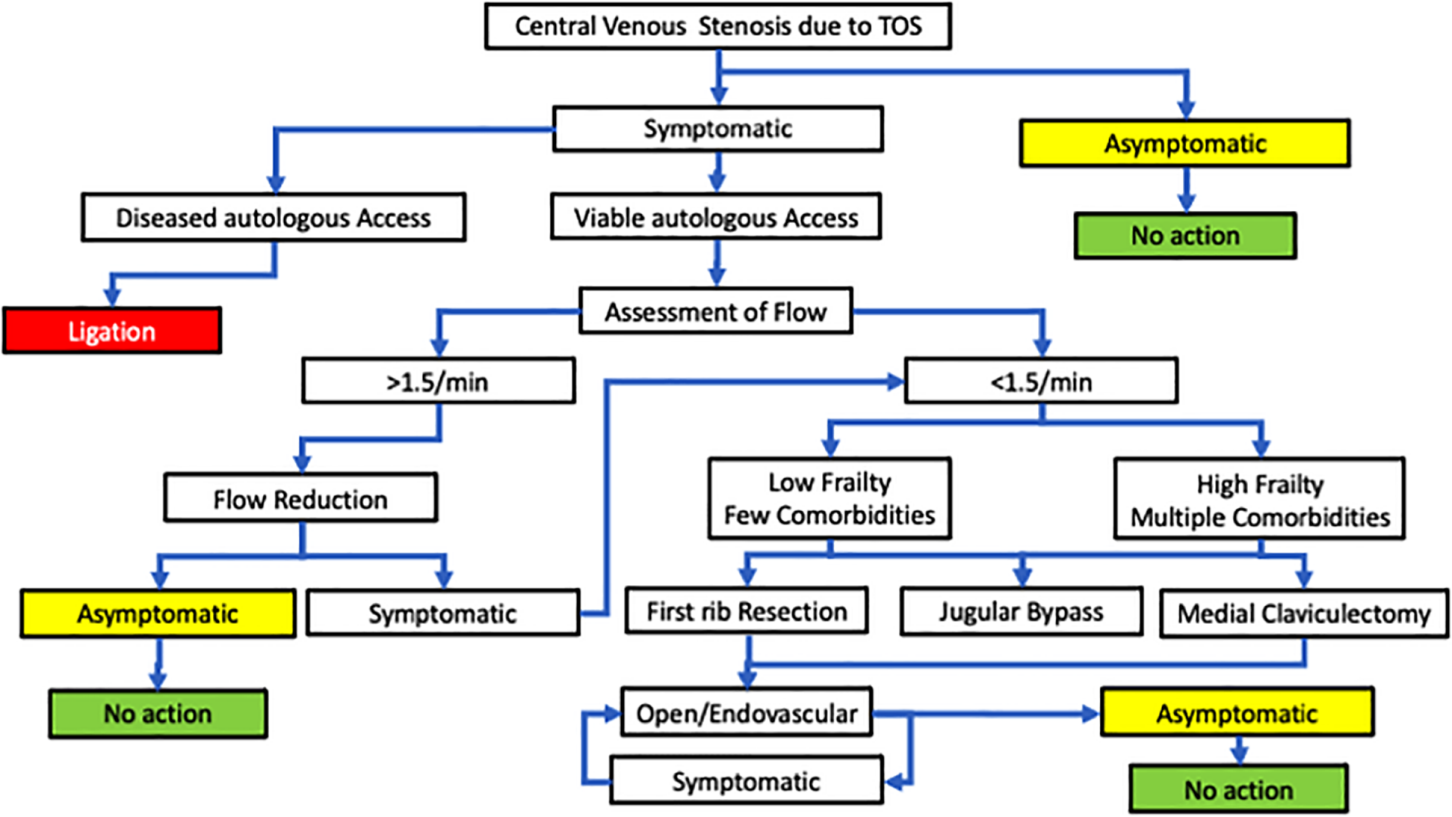
An algorithm to treat central venous stenosis due to thoracic outlet syndrome.

The indications for intervention are symptomatic arm swelling and arm pain or AV access malfunction. The treatment algorithm is shown in Figure 1.

## Interventions

### Flow reduction techniques

In good-quality vascular access circuits on the same side as the symptomatic HDTOS, the flow should be assessed and categorized as high flow (>1.5 liters/min) or acceptable flow (<1.5 liters/min). Access circuits with high flow (>1.5 liters/min) should be considered for a flow reduction procedure to achieve a flow of less than 1.5 L/min (8). These procedures can be banding or arterial proximalization. Simply adjusting the flow can achieve relief of the major symptoms of venous congestion and allow collateral outflow to develop and offset any CCJ-induced stenosis. This negates the need for further open or endovascular intervention in the culprit area at the CCJ.

### Endovascular interventions

The commonest direct intervention for TOS compression and CCJ-induced stenosis is the use of various endovascular techniques (angioplasty with conventional, high-pressure cutting or drug-eluting balloons with or without open cell or covered self-expanding stent placement) to correct the stenosis encountered during an intervention for malfunction arteriovenous access/ occlusion or a subclavian DVT. Unfortunately, there is no specific data on treated CCJ-associated stenosis in the current clinical data sets because all central venous stenoses are combined in the reporting of the venous intervention. Outcomes for endovascular intervention show that primary angioplasty has an overall patency of 48% to 100%, while outcomes for conventional stenting range from 78% to 100%. Reintervention rates in these patients range from 2 to 2.7 per patient per year. Covered stent grafting has better outcomes (9). There is a growing opinion that there is a high incidence of recurrence at the CCJ area and that there is a higher stent fracture rate leading to occlusion and loss of outflow from the arm. Following decompression, the use of open cell and covered stents to reopen a stenosed segment is reported (10–16).

### Catheter-based interventions

The Hemodialysis Reliable Outflow (HeRO) device is a vascular access system consisting of a large bore central venous catheter that allows a bypass of central venous stenoses or occlusions and a connector that allows connection with arteriovenous autologous, allograft, or prosthetic access in the arm (17). This device has been used to bypass hdTOS stenoses and occlusions without resorting to thoracic outlet decompression. Functional and patency rates of the HeRO graft have been reported to be comparable to arteriovenous grafts without central venous disease (18).

### Subclavian vein reconstruction or bypass

When there is no option to treat the CCJ and there is reluctance to place a HeRO graft, bypass of the obstructing venous lesions has been proposed.

*Jugular vein bypass:* A alternative option to TOS decompression is to bypass the subclavian vein with a jugular vein turn down, a venous or prosthetic conduit bypass from the axillary vein to the jugular vein on the same side (19, 20).
*Central venous bypass.* If the option of jugular vein bypass does not exist, central venous bypass has been proposed with the conduit running from the axillary vein to the right atrium. The conduit can be paneled saphenous vein, allograft vein, or prosthetic conduit. This is generally reserved for patients with good physiological reserve (9, 20).

During decompression procedures the surgeon has the option to patch the subclavian vein or replace it with a venous conduit if there is no desire to simply use a covered endovascular stent.

### Decompression techniques

A newer therapeutic option has been proposed to counter the issues of stenosis at the CCJ. There are several approaches to effectively remove the extrinsic compression of the subclavian vein, one based on the removal of the first rib and the second based on the removal of the clavicular head and first third of the clavicle. Once the extrinsic compression is corrected, the surgeon has the option to patch the subclavian vein or replace it with a venous conduit or to intervene endovascularly to correct the associated intrinsic mural disease.

*Trans-axillary Approach:* In the trans-axillary approach, the thoracic outlet is entered using a transverse incision in the axillary region. The neurovascular structures are dissected free and the first rib is exposed. The first rib is then dissected free of its muscular attachments, cleaned, and transected anterior to the vein and posterolateral to the artery. Venolysis of the subclavian vein is performed to ensure maximum venous dilation after the space is cleared. Additional dissection allows for the subclavius muscle and tendon to be debulked (21). These maneuvers allow for the CCJ area dimensions to greatly increased and eliminate the anatomic barriers seen in hdTOS.
*Anterior Approach:* The infraclavicular approach, often combined with the supraclavicular approach, is the most popular approach to gain access to the thoracic outlet and its venous structures. Using the infraclavicular approach, one can divide the subclavius and scalene muscles, resect the first rib and achieve venolysis of the subclavian vein (21). While the supraclavicular approach to the thoracic outlet remains the least popular surgical approach for venous thoracic outlet pathology, it can facilitate division of the scalene muscles and any fibrotic bands and resection of cervical ribs (22).
*Medial Claviculec*tomy: In patients considered unsuitable for 1st rib resection, removal of the medial portion of the clavicle to unroof the area of the CCJ has been advocated. To achieve the goal of freeing up the CCJ area of the thoracic outlet, an incision is made along the clavicle and the clavicle is divided two thirds of the way out laterally, the claviculosternal junction disarticulated, and all associated muscles are divided and resected (23). Once the clavicle is removed, the vein up to the confluence with the jugular vein and into the innominate vein can be accessed, and an extensive venolysis can be performed (12).
*Sternoclavicular rotation*: A variant on the medial claviculectomy is the sternoclavicular rotation. In this case, exposure is obtained by performing a first interspace partial sternotomy, maintaining integrity of the sternoclavicular joint and rotating it upward, which permits subclavian vein venolysis and reconstruction afterward (24).
*Video-assisted thoracic surgery and robotic assisted first rib resection*. Video assisted thoracic surgery has been used to remove the first rib by an intrathoracic approach. It provides excellent visualization of the thoracic outlet with the goal of removing the first rib (25–27).

### Current outcomes with TOS decompression in HD patients

There is very limited data available from very few centers (7 reports; 2011–2022) evaluating surgical thoracic outlet decompression for subclavian vein stenosis at the CCJ in an attempt to salvage a threatened hemodialysis access (Table 1). There have been seven clinical reports series that have been published (10–16). One hundred and twenty-seven patients (52% male) have undergone open thoracic outlet decompression for hdTOS using multiple modalities (Anterior Approach:48%; Partial claviculectomy: 29%; TransAxillary: 23%). Seventeen percent did not require a concomitant intervention, while the remaining 83% underwent various open (Patch angioplasty: 16%; Open bypass: 5%) or endovascular interventions (Uncovered Stent:44%; Angioplasty: 12%; Covered Stent: 10%) to secure a patent vein. Following these combined interventions, 1-yr median primary patency was 37%, while median 1-yr secondary patency was 84%. Overall median access functionality at 1-yr was 85%.

**Table 1:** TOS decompression reports.

A												
				!st Rib Procedure			EV interventions				Open interventions	
Year	Author	Reference	n	TransAxillary	Anterior Approach	Partial claviculectomy	No EV intervention	Angioplasty	Uncovered Stent	Covered Stent	Patch Angioplasty	Bypass
2011	Glass	10	10	6	0	4	2	5	3	0	0	1
2015	Illig	11	24	0	21	3	0	0	1	0	0	2
2019	Auyang	12	21	0	0	21	0	4	11	6	21	0
2019	Wooster	13	34	0	31	5	6	0	34	0	0	0
2019	Edwards	14	4	0	0	4	0	0	0	0	0	4
2021	Lim	15	19	18	0	0	10	7	3	0	0	0
2022	Uceda	16	15	5	10	0	0	0	7	8	0	0
B												
				Outcomes			
Year	Author	Reference	F/U Months	MACE	1-yr Primary	1-yr Secondary	1-yr Access functionality
2011	Glass	10	7	0%	–	–	–
2015	Illig	11	10	0%	40%	85%	85%
2019	Auyang	12	17	0%	28%	84%	68%
2019	Wooster	13	11.9	6%	NR	NR	NR
2019	Edwards	14	30	25%	NR	NR	NR
2021	Lim	15	39	0%	42%	69%	93%
2022	Uceda	16	35	0%	33%	NR	NR

EV, endovascular; F/U, Follow Up; NR, not reported; MACE, Major Adverse Cardiovascular Events.

### Current guidelines

The current Kidney Disease Outcomes Quality Initiative (K-DOQI) guidelines from the National Kidney Foundation currently do not recommend intervention for physiological compression or mild subclavian vein stenosis (28). In patients with moderate to severe symptoms where there is associated ineffective dialysis, intervention is recommended. Endovascular balloon angioplasty is recommended as first-line therapy, with intraluminal stenting reserved for (1) acute elastic recoil >50% after percutaneous transluminal angioplasty (PTA) or (2) recurrent stenosis within 3 months. No recommendation is currently made on the type of stent to use, Thoracic outlet decompression has not been discussed nor is it recommended as standard of care in the current guidelines.

## Conclusion

hdTOS represents a complex and evolving therapeutic conundrum for the dialysis community and additional clinical investigations to establish robust algorithms are required. Currently, hdTOS represents a critical issue for further investigation and decompression of TOS should only be performed in carefully selected patients where the risk benefit analysis is appropriate and should only be carried out in centers with substantial experience in advanced decompression of the thoracic outlet.

## Author contributions

Study design: MGD and JPH. Obtain data: MGD and JPH. Statistical analysis: MGD and JPH. Data interpretation: MGD and JPH. Manuscript draft: MGD and JPH. Critical revision: MGD and JPH. All authors contributed to the article and approved the submitted version,

## Conflict of interest

The authors declare that the research was conducted in the absence of any commercial or financial relationships that could be construed as a potential conflict of interest.
